# The role of sociodemographic factors on the acceptability of digital mental health care: A scoping review protocol

**DOI:** 10.1371/journal.pone.0301886

**Published:** 2024-04-26

**Authors:** Nagi Abouzeid, Shalini Lal

**Affiliations:** 1 School of Rehabilitation, University of Montréal, Montréal, Québec, Canada; 2 Youth Mental Health and Technology Lab, University of Montréal Hospital Research Centre, Montréal, Québec, Canada; 3 Douglas Research Centre, Montréal, Québec, Canada; King Saud University Medical City, SAUDI ARABIA

## Abstract

**Introduction:**

Many individuals experiencing mental health complications face barriers when attempting to access services. To bridge this care gap, digital mental health innovations (DMHI) have proven to be valuable additions to in-person care by enhancing access to care. An important aspect to consider when evaluating the utility of DMHI is perceived acceptability. However, it is unclear whether diverse sociodemographic groups differ in their degree of perceived acceptability of DMHI.

**Objective:**

This scoping review aims to synthesize evidence on the role of sociodemographic factors (e.g., age, gender) in the perceived acceptability of DMHI among individuals seeking mental health care.

**Methods:**

Guided by the JBI Manual of Evidence Synthesis, chapter on Scoping Review, a search strategy developed according to the PCC framework will be implemented in MEDLINE and then adapted to four electronic databases (i.e., CINAHL, MEDLINE, PsycINFO, and EMBASE). The study selection strategy will be piloted by two reviewers on subsets of 30 articles until agreement among reviewers reaches 90%, after which one reviewer will complete the remaining screening of titles and abstracts. The full-text screening, data extraction strategy, and charting tool will be completed by one reviewer and then validated by a second member of the team. Main findings will be presented using tables and figures.

**Expected contributions:**

This scoping review will examine the extent to which sociodemographic factors have been considered in the digital mental health literature. Also, the proposed review may help determine whether certain populations have been associated with a lower level of acceptability within the context of digital mental health care. This investigation aims to favor equitable access to DMHI among diverse populations.

## Introduction

Many living with mental health complications face barriers when attempting to access mental health services (e.g., limited availability of services, costs) [1]. To bridge the existing treatment gap, digital mental health innovations (DMHI) have proven to be valuable additions to standard treatment due to their ability to enhance access to care for those living with precarious mental health conditions (e.g., anxiety, depression) [2–7]. DMHI are digital health technologies used to assess, prevent, or treat mental health difficulties [8]. An important aspect to consider when evaluating the utility of DMHI is perceived acceptability. Acceptability broadly refers to the way individuals perceive, and experience a given digital innovation; it is also a significant predictor of an intervention’s effectiveness [9].

Over the past decade, reviews of the literature, including systematic reviews, have been conducted to synthesize the available evidence on acceptability in the context of digital health care [10–16]. However, these reviews focused on specific populations (e.g., adults with depression and college students) [10–12,14,16], included interventions beyond those relating to mental health (e.g., sexual health) [14], and did not report on associations between sociodemographic characteristics and perceived acceptability [10–15].

As demonstrated, there have been limited efforts to systematically investigate the role of sociodemographic characteristics on the perceived acceptability of DMHI among diverse populations. Specifically, it is unclear whether there are any discernible trends of lower or higher levels of perceived acceptability among various sociodemographic groups within the context of digital mental health care. Such a synthesis is important, considering the impact of perceived acceptability in sustaining user engagement [9]. Research has shown that a greater level of engagement with DMHI is significantly associated with improved mental health outcomes [17]. Therefore, to enhance user engagement and maximize therapeutic outcomes, it is important to gain a deeper understanding of acceptability and its associated factors within the context of digital mental health care.

The proposed scoping review (registered in OSF: osf.io/dvr53) intends to synthesize research that reports on associations between sociodemographic characteristics and the perceived acceptability of DMHI. In the context of this review, the term “association” extends beyond its statistical sense and refers to the presence of qualitative patterns of association between specific sociodemographic groups, and their perceived acceptability of DMHI. This review will help to determine whether a more systematic investigation on the topic, such as a meta-analysis, is needed. Moreover, we may find that certain sociodemographic characteristics have been inadequately accounted for in the development and delivery of DMHI. This undertaking is aligned with a broader objective aiming to ensure underserved populations have access to DMHI that they perceive as acceptable.

Numerous factors informed the decision to opt for a scoping review as opposed to another type of review. First, a scoping review can be used to investigate broad topics of inquiry without requiring a quality assessment of the studies that are discussed [18]. In contrast, a systematic review will generally seek to answer a more specific research question and will require an assessment of the methodological rigor of the studies included [18]. Considering these differences, a scoping review is a more suitable option due to the broad scope of the proposed investigation. Moreover, it is difficult to evaluate the feasibility of conducting a full systematic review without first conducting a preliminary mapping of the literature that would help justify such an undertaking.

### Objective and review questions

The primary objective of the proposed review is to synthesize evidence on the role of sociodemographic characteristics on the degree of perceived acceptability of DMHI. The research questions listed below have been informed by the primary objective of the proposed scoping review as well as the population, concept, and context (PCC) framework proposed by the Joanna Briggs Institute (JBI) manual of Evidence Synthesis [19]:

1. What are the different sociodemographic factors that have been considered in the evaluation of perceived acceptability of DMHI?
2. Which sociodemographic factors have been positively, negatively, or not associated with the perceived acceptability of DMHI?
3. Which DMHI have been positively, negatively, or not associated with perceived acceptability among specific sociodemographic groups? (Please see the *Study Selection and Extraction* section below for more details on how the various DMHI will be categorized)

### Eligibility criteria

The PCC framework proposed by the Joanna Briggs Institute (JBI) manual of Evidence Synthesis [19] will inform the inclusion and exclusion criteria (Table 1) of the proposed scoping review.

**Table 1:** Inclusion and exclusion criteria.

**Inclusion Criteria**
• Human participants receiving digital mental health care. • Reports on the age, gender, sexual orientation, race, education level, or health status of participants. • Relative acceptability among subgroups is reported, absolute acceptability per subgroup is reported, or the study sample is homogeneous for at least one sociodemographic factor of interest. • The study reports on the perceived acceptability, utility, or ease of use of digital mental health innovations. • Acceptability is evaluated retrospectively. • Acceptability is evaluated through quantitative methods. • Mental health care is partly or completely delivered through technology. • Digital innovations that primarily aim to enhance psychological, emotional, or social well-being. • Articles written in French or English • Articles published between January 2013 and June 2023 • Primary Sources
**Exclusion Criteria**
• Clinicians and/or researchers evaluating the acceptability of digital mental health care. • Acceptability is measured using objective measures (e.g., adherence, use). • Qualitative studies

#### Population

The proposed scoping review will include studies conducted with human participants. To be considered for inclusion, participants must receive mental health care through the use of DMHI. The proposed scoping review will consider six different sociodemographic dimensions: age (e.g., youth, adult, and elderly), race (e.g., Arabic, Asian, and White), gender identity (e.g., male, female, and genderfluid), sexual orientation (e.g., heterosexual, homosexual, and bisexual), highest level of education completed (e.g., primary school, high school, and bachelor’s degree), and health status (e.g., depression, schizophrenia).

Moreover, for studies to be considered eligible, they must either report on the relative acceptability among participants (e.g., women demonstrating a higher level of acceptability compared to men) or the absolute acceptability (e.g., women rating the innovation with a 4 out of 5 in terms of perceived acceptability). Regarding absolute acceptability, we will consider studies as eligible if the perceived acceptability of subgroups within the larger sample is reported or if the study sample is homogeneous for at least one of the sociodemographic factors of interest.

#### Concept

In this scoping review, the concept of interest is acceptability, a multidimensional notion with various definitions [20]. Acceptability is broadly defined as the way individuals perceive and experience a given digital health innovation [9]. More specifically, two important factors contributing to the perceived acceptability of a given technology are perceived usefulness and ease of use [21]. To be considered for inclusion in the proposed review, the studies must focus on examining the perceived acceptability (as defined by the author(s) of the studies being reviewed), utility, or ease of use of DMHI. Moreover, to be considered for inclusion in the proposed review, acceptability must be evaluated retrospectively. Therefore, studies that evaluate the prospective acceptability of DMHI will not be included in the proposed review. For instance, studies that assess the acceptability of a digital technology among potential users, who have not yet engaged with the innovation, will be excluded from the proposed review. In addition, to be considered for inclusion, acceptability must be evaluated through quantitative methods (e.g., questionnaires and scales), these may or may not have been validated. Studies that examine the perceived acceptability of users entirely through qualitative methods (e.g., focus group discussions, open-ended questions) will not be considered for inclusion in the proposed review.

Moreover, the concept of acceptability is person-centered. Therefore, any studies that define or assess acceptability without considering the perspective of the user will be excluded from the proposed review. For instance, any study that examines the theoretical utility of a technology from the perspective of clinicians as opposed to the perceived utility of a technology from the perspective of users will be excluded from the proposed review. The purpose of these eligibility criteria is to establish clear boundaries for the broad concept of acceptability and to ensure a consistent conceptualization across the included studies. Furthermore, the definition of acceptability, as proposed in each of the included studies, will be reported to ensure that readers have a clear understanding of the concept presented.

#### Context

In this scoping review, the focus will be on DMHI delivered in both clinical settings (e.g., hospitals) and non-clinical settings (e.g., community). DMHI refer to the use of technology in the assessment, prevention, and treatment of mental health difficulties [8]. To be considered for inclusion, the mental health intervention must be delivered digitally, such as through a computer, smartphone, or wearable device. There will be no studies excluded based on the specific digital care medium used to deliver the mental health intervention. Some examples of DMHI include, but are not limited to, internet-based cognitive behavioral therapy, chatbots for mental health support, smartphone applications that support ecological momentary assessment, web-based psychotherapeutic platforms, psychosocial assessments through video conferencing solutions, and exposure therapy assisted by virtual reality [22–24].

Interventions delivered in a blended format (i.e., involving in-person and digital components) will only be considered for inclusion if the acceptability of the digital component is assessed independently. For example, studies will not be considered for inclusion if they report a global acceptability score for an intervention comprised of three in-person therapy sessions and two online therapy sessions. However, studies that report on the acceptability of the digital component (i.e., online sessions) independently will be considered for inclusion.

To delimit the broader concept of mental health, interventions that primarily aim to enhance the psychological (e.g., cognitive biases), social (e.g., isolation), and emotional (e.g., sadness) well-being of an individual will be considered for inclusion. Moreover, interventions that target sleep difficulties within mental health populations will be considered for inclusion.

The proposed review will not include DMHI that address both mental health and physical health (e.g., exercise, nutrition), sexual health (e.g., HIV prevention), cognition (e.g., attention, memory), or pain management where the primary objective is not focused on optimizing mental health. For instance, a digital intervention that aims to reduce rumination while promoting physical activity to reduce the risk of cardiovascular diseases would not be included in the proposed review. If this review were to include interventions with an emphasis on health targets beyond those related to mental health, it would be difficult to determine whether the role of sociodemographic factors is associated with mental health care, physical health care, or both. Moreover, digital interventions that aim to improve medication adherence (e.g., SSRI, SNRI) to enhance mental health outcomes will be considered for inclusion. However, digital interventions that aim to improve medication adherence to enhance physical health outcomes (e.g., HIV, diabetes) would not be considered for inclusion.

Any study that does not satisfy the aforementioned eligibility criteria will be excluded from the proposed scoping review.

#### Types of sources of evidence

Moreover, there are other notable eligibility criteria unrelated to the PCC framework. First, only materials available in French and English will be included in the proposed scoping review. Second, to ensure the recency and relevance of the synthesized materials, only materials disseminated between January 2013 and June 2023 will be included in the proposed review; this will help ensure that the findings reported are relevant to the present societal context. Any secondary sources (e.g., meta-analysis and systematic reviews), study protocols, commentary or opinion letters, or non peer-reviewed materials will be excluded from the proposed review. Studies with a purely qualitative design will also be excluded from the proposed review. Any study that does not satisfy the aforementioned eligibility criteria will be excluded from the proposed review.

## Methodology

The proposed scoping review will be guided by the recommended methodology put forth in the JBI Manual of Evidence Synthesis [19] and the PRISMA-P checklist (S2 File) (http://www.prisma-statement.org/Extensions/Protocols.aspx).

### Sources of evidence

The information sources that will be used to identify materials pertinent to the proposed review have been selected to ensure the review is as extensive and comprehensive as reasonably possible on the topic of inquiry. Four electronic databases will be examined using distinct search strategies (i.e., PsycInfo through Ovid, MEDLINE through Ovid, CINAHL through EBSCO, and Embase through Ovid). Moreover, the reference list of secondary sources identified as relevant to the topic of inquiry will be examined.

### Search strategy

Initially, A1 developed the concept plan for the search strategy in consultation with A2. A search strategy was then developed by A1 and later refined in collaboration with A2 and an experienced librarian at the University of Montreal. As a first step in the development of the search strategy, a preliminary search of MEDLINE and CINAHL was conducted. These databases were selected due to their extensive assortment of literature on health research. This was then followed by the selection of keywords identified in the abstracts and titles of retrieved papers along with indexed terms used to categorize the publications. The keywords and index terms were considered to inform the development of a complete search strategy for MEDLINE (S1 File).

The search strategy incorporates three topics of interest, which are acceptability, digital technology, and mental health. For acceptability, one indexed keyword heading (i.e., Patient acceptance of healthcare) (line 1) was searched along with three sets of search terms (line 2, 3 and 4) relating to acceptability (e.g., client acceptance, perceived utility). The indexed keyword and the first three sets of listed terms were then linked with the Boolean operator “OR” (line 5).

For the topic of digital technology, 10 indexed keyword headings (e.g., Internet-based interventions, mobile applications) were searched (lines 6,7,8, 9,10,11, and 12) along with one set of search terms incorporating many concepts related to digital technology (e.g., digital, bots) (line 13). This set of search terms was complemented by a list of keywords proposed by Lal et al. [23] in a systematic review pertaining to the priority afforded to technology in government-based mental health strategy documents. The indexed keyword headings relating to digital technology along with the related set of search terms were then linked with the Boolean operator “OR” (line 14).

For the topic of mental health, four indexed keyword headings were searched (e.g., mental health, mental disorders) (lines 15,16,17, and 18) along with five sets of search terms relating to mental health (e.g., low mood, mania, bereavement) (Line 19,20,21,22, and 23). These initial sets of search terms were bonified by the mental health disorders listed in the ICD-11 [25] and DSM-V [26] along with their associated symptoms. The indexed keyword headings relating to mental health along with the sets of related search terms were then linked with the Boolean operator “OR” (line 24). Lines 5, 14, and 24 were then combined using the Boolean operator “AND” to identify relevant citations that will be considered for inclusion in the proposed scoping review. The search was then further limited by year of publication (i.e., 2013 to current) and language (i.e., French and English). With the guidance of an experienced librarian, A1 will adapt and implement the MEDLINE search strategy to three other electronic databases (i.e., PsycINFO, CINAHL, and Embase).

### Study selection and extraction

The inclusion and exclusion criteria outlined above have been established to guide the selection of studies that are concordant with the primary objective of the proposed scoping review. The citations obtained from the literature search will be imported into Covidence, a software used to assist in evidence synthesis. Covidence has the ability to extract citations, remove duplicates, and allows for a multiphase review of citations by up to two reviewers [27].

First, as proposed in the JBI Manual of Evidence Synthesis [19], the study selection strategy for titles and abstracts will be piloted on a random subset of citations to ensure that reviewers have an adequate understanding of the eligibility criteria and selection process. The screening process will be guided by the eligibility criteria of the proposed review. To begin, a subset of 30 citations will be selected from the pool of obtained studies. These articles will then be screened independently by two reviewers. Once completed, the reviewers will discuss any discrepancies in the perceived eligibility of studies to determine if the eligibility criteria require any further clarifications. This process will be repeated until the eligibility agreement between reviewers reaches 90%. Once achieved, the complete screening of citations by A1 will begin. The citations that do not contain sufficient information in the titles and abstracts to assess eligibility or those that are deemed eligible will then undergo full-text screening.

Once the screening of titles and abstracts is completed, a three-step process will be used to determine which studies will be included in the proposed scoping review. First, the full-text screening strategy will be piloted on 15 citations selected from the pool of obtained studies. These articles will then be screened independently by two reviewers. Once completed, the reviewers will discuss any discrepancies in the perceived eligibility of studies to determine if the eligibility criteria require any further clarifications. This process will be repeated until the eligibility agreement between reviewers reaches 90%. Once achieved, the complete screening of full-text citations by A1 will begin. A second reviewer will then validate all the studies deemed eligible by A1. If discrepancies arise in the process of eligibility assessment, A1 will meet with the second reviewer to reach a final consensus. If a consensus cannot be reached, a third reviewer will be consulted.

Once eligible articles have been selected, the process of data extraction and charting will begin. To facilitate the process of extracting data and ensure its validity, a legend will be developed. This legend will clearly identify and describe the information that should be included in each category of the charting tool. The categories and their corresponding data will be organized in an Excel sheet. As a first step, we will consider data categories listed in the JBI Manual of Evidence Synthesis [19]. These include basic descriptive information found within citations (i.e., title, Digital Object Identifier (DOI), author(s), and year of publication), as well as the country of origin, sample size, study design, study objective(s), and key outcomes. Key outcomes will be identified based on their degree of responsiveness to the research questions put forth by the proposed scoping review.

Furthermore, these categories will be further complemented by data relating to the PCC framework and research questions of the proposed review. Considering the population component of the PCC framework, we will extract data relating to the study sample, including their health status (e.g., diagnosed with a major depressive disorder) and reported sociodemographic characteristics (e.g., age, gender, race, sexual orientation). In relation to the concept component of the PCC framework, the conceptualization of acceptability (e.g., perceived ease of use) put forth in the articles along with the method(s) used to evaluate acceptability (e.g., self-report questionnaire) will be extracted. Lastly, considering the context component of the PCC framework, we will extract information on the type of DMHI evaluated (e.g., ICBT), the purpose of the innovation (e.g., assessment), its mental health target (e.g., anxiety), and the technology used (e.g., smartphone).

The data charting tool that will be used is subject to an Iterative process. As data are extracted, additional categories may emerge that warrant inclusion in the charting tool. As recommended in the JBI Manual of Evidence Synthesis [19], the data charting tool will be piloted by extracting data from 15 studies. This process will help ensure that the data charting tool is comprehensive and easy to use. Two independent reviewers will be involved in the pilot data extraction process. Any discrepancies that may arise during the data extraction or categorization process will be discussed by two reviewers to reach a final consensus. If consensus cannot be reached, a third reviewer will be consulted to examine and resolve any identified discrepancies. Once completed and sufficient concordance among extracted data has been achieved, A1 will begin the data extraction process for the complete list of eligible citations. The extracted data will then be validated by a second reviewer.

### Data synthesis and presentation

First, a table will be used to represent the various DMHI that have had their acceptability evaluated through a consideration of sociodemographic characteristics. This table will include data on the various types of DMHI (e.g., ICBT), their mental health targets (e.g., anxiety), their purpose (e.g., assessment), and the technology used (e.g., smartphone). Secondly, a table will be presented to illustrate the different sociodemographic characteristics that have been considered in the evaluation of acceptability of DMHI. The extracted data will be summarized and presented using descriptive statistics (e.g., frequency and percentages).

Finally, key findings on the role of sociodemographic characteristics on the perceived acceptability of DMHI will be presented in tables, including information on whether the association is positive (i.e., high perceived acceptability), neutral (i.e., no association) or negative (i.e., low perceived acceptability). These findings will be categorized based on the technology examined (e.g., smartphone app), sociodemographic factor(s) of interest (e.g., age), and whether they are relative (e.g., men demonstrated a higher level of perceived acceptability than women for this technology) or absolute (e.g., men demonstrate a high level of perceived acceptability for this technology).

For absolute acceptability, the cut-off scores reported in the literature for the various measures used to measure acceptability across studies will be used to indicate whether the reported scores represent high or low acceptability. If such scores are not available, the acceptability scores that have been evaluated using the same measures across studies will be contrasted. For studies that utilized ad-hoc measures to examine the perceived acceptability of users, the author’s interpretation of these scores will be reported in the proposed review. The approach used to interpret the various scores will be finalized depending on how acceptability is being measured across the included studies.

Furthermore, as the data extraction strategy is piloted, additional findings may be reported based on their relevance to the inquiries put forth in the proposed scoping review. Moreover, throughout the screening and extraction processes, notable patterns may emerge across studies, which may not have been specified in this protocol. These may include methodological limitations (e.g., many studies did not report on the socio-demographics of study dropouts), the overrepresentation of specific digital mental health innovations (e.g., virtual reality), or their mental health targets (e.g., depressive symptoms).

Moreover, depending on the volume of findings uncovered (i.e., over 100 studies included), it may be necessary to use additional forms of visual representation, such as graphs and Venn diagrams. These may be used to provide readers with a complementary depiction of findings to facilitate greater comprehension.

## Limitations

To the authors’ knowledge, the proposed scoping review will be the first of its kind to synthesize and report on the associations between various sociodemographic characteristics and the perceived acceptability of DMHI. However, such an undertaking is not without limitations.

First, this review will not evaluate the methodological rigor of the included studies. Such an undertaking would help determine the quality of the evidence on the role of sociodemographic characteristics on the acceptability of DMHI. These findings may then potentially be used to demonstrate the necessity for culturally adapted DMHI and guide their future development. To alleviate the impact of this limitation, the study design (e.g., randomized controlled trial, case study) of the included citations will be reported.

Second, considering the diversity of terminology employed in the field of digital mental health care [28], it is possible that the proposed search strategy does not capture all studies that warrant inclusion in the proposed scoping review. To address this limitation, A1 and A2 collaborated with an experienced librarian to develop a search strategy that will be adapted and implemented in four electronic databases. The search strategy was also supplemented with search terms put forth in a systematic review by Lal et al. [23]. However, given the rapid evolution of technological advancements, eligible studies may still go undetected and hence not be included in the proposed scoping review. Moreover, the decision to omit related terms such as “satisfaction” and “preference” from the search strategy, while helping with feasibility and limiting the scope of this review to the specific concept of acceptability, also stands as a potential limitation of the proposed review.

Third, after conducting pilot testing of the screening strategy, only one reviewer will be responsible for assessing the eligibility of abstracts. However, mitigation strategies have been implemented to minimize the impact of this limitation. For instance, at the stage of full-text screening, a second reviewer will validate all the studies deemed eligible by A1.

Finally, based on the volume of results obtained through a preliminary screening of the literature, non peer-reviewed materials (e.g., Theses) will not be considered for inclusion in the proposed review.

## Conclusion

Despite its limitations, the proposed scoping review will aim to map the available scientific evidence pointing to associations between sociodemographic characteristics and perceived acceptability of DMHI. These findings will allow us to determine the extent to which sociodemographic characteristics are being considered in the digital mental health literature along with their reported associations with perceived acceptability. Specifically, the proposed review will enable us to determine whether certain sociodemographic groups have frequently been linked with lower perceived acceptability of DMHI. This investigation represents a first step to ensure that diverse populations can access and engage with DMHI that they perceive as acceptable.

Moreover, the proposed scoping review will provide insights for future research on ways to assess and report on the role of sociodemographic characteristics when evaluating the perceived acceptability of DMHI. This synthesis of knowledge can also serve as a justification and provide guidance for conducting a systematic review and meta-analysis on the topic of inquiry, within which the methodological rigor of included studies will be examined. The findings from this type of review may serve as evidence to guide the development and delivery of culturally tailored DMHI.

## Supporting information

S1 File Detailed search strategy for MEDLINE. (DOCX)

S2 File PRISMA-P checklist. (DOCX)
