# Supplementary material for: The role of sociodemographic factors on the acceptability of digital mental health care: A scoping review protocol
Source: PLoS One. 2024 Apr 26;19(4):e0301886. doi: 10.1371/journal.pone.0301886 (PMC11051634; doi:10.1371/journal.pone.0301886)
Supplement: S1 File — (DOCX) [file pone.0301886.s001.docx]

1 "Patient Acceptance of Health Care"/ 54751

2 acceptability.ab,kf,ti. 52411

3 ((Patient* or Client*) adj4 Acceptance).ab,kf,ti. 7525

4 ("Perceived Utility" or usefulness or "ease of us*").ab,kf,ti. 159794

5 1 or 2 or 3 or 4 266376

6 exp Internet-Based Intervention/ 1115

7 exp Text Messaging/ 4479

8 exp Mobile Applications/ 11406

9 exp telemedicine/ or exp telerehabilitation/ 44574

10 exp Digital Technology/ 689

11 exp Telecommunications/ or exp Videoconferencing/ or exp Remote Consultation/ 123931

12 exp Telenursing/ 250

13 (digital or online or virtual or remote or "web-based" or "internet-based" or "mobile-based" or "mobile app*" or Telemedi* or "tele-medi*" or telepsych* or "tele-psych*" or Telerehabilitation or "tele-rehabilitation" or teleconferenc* or "tele-conferenc*" or Teleconsult* or "tele-consult*" or "telemental health" or "tele-mental health" or "wearable device*" or "m-health" or mhealth or "mobile health" or "mobile device*" or "tele-therap*" or teletherap* or telehealth or "tele-health" or "Text messag*" or SMS or "video-assisted" or "video-health" or "video psych*" or "video therap*" or bots or ICBT or gamifi* or Ehealth or "e-health" or teleassist* or "tele-assist*" or "tele-care" or telecare or "e-consult*" or econsult* or "tele-communication*" or telecommunication* or telephone* or "mobile phone*" or "cell phone*" or "artificial intelligence" or avatar* or chat* or "smart phone*" or smartphone* or "smart watch*" or smartwatch* or computer* or laptop* or cyber* or "portable device*" or "e-mental" or emental or website* or technolog* or ereferral* or "e-referral*" or "tele-nursing" or telenursing or videoconferenc* or "video-conferenc*").ab,kf,ti. 1684426

14 6 or 7 or 8 or 9 or 10 or 11 or 12 or 13 1732108

15 exp Mental Health/ 61027

16 exp Mental Health Services/ 105354

17 exp Mental Disorders/ 1430026

18 Psychological Trauma/ 1860

19 ((mental* or psych*) adj2 (health or wellbeing or well-being or disorder* or ill* or disease* or issue* or disturbance* or problem* or difficult*)).ab,kf,ti. 445348

20 (anxiet* or anxious or depress* or stressor* or suicid* or "self-harm" or bereavement or obsession* or compulsi* or dissociat* or rumination* or worr* or "binge-eating" or insomnia* or hypersomnolence or "sleep-walking" or somnambulism or "sleep terror*" or "night terror*" or paranoi* or hallucin* or delusion* or mania or hypomani* or overeating or "over eating" or "self-esteem" or guilt or nervousness or apathy or avolition or anhedonia or alogia or asociality or hopelessness or worthlessness or grief or "low mood").ab,kf,ti. 1124075

21 (psycho* or schizo* or anorexi* or bulimi* or agoraphobia or sociopath* or "body dismorphi*" or depersonalization or derealization or dysthymi* or "gender dysphori*" or hysteri* or melancholi* or orthorexia* or "panic attack*" or phobi* or catatoni* or dysthymi* or mutism or trichotillomani* or PTSD or pica or enuresis or encopresis or pyromania or kleptomani* or hebephreni* or paraphreni* or neurosis or neurastheni* or "pathological fire-setting" or "pathological stealing" or sadis* or masochis* or "ego-dystoni*" or egodystoni* or "body integrity dysphori*" or "delayed ejaculation" or "premature ejaculation" or "sexual dysfunction*" or hypochondria* or bipolar or "psychological trauma" or "emotional trauma").ab,kf,ti. 1095177

22 ((adjustment or gambling or eating or conduct* or "oppositional defiant" or personality or dissociative or feeding or mood or neurotic or paraphilic or "substance-related" or cyclothymi* or cycloid or panic or hoarding or "hair-pulling" or excoriation or "skin-picking" or attachment or "disinhibited social engagement" or "somatic symptom" or conversion or factitious or "food intake" or elimination or "sleep wake" or "sleep arousal" or "nightmare*" or behavi* or "impulse-control" or antisocial or borderline or histrionic or narcissistic or avoidant or dependen* or paraphilic or voyeuris* or exhibitionis* or frotteur* or pedophili* or fetish* or transvestic or trance or possession or affective or somatoform or somatization or dissocial or "emotionally unstable" or anankastic or eccentric or hatlose or immature or "passive-agressive" or habit or impulse or "intermittent explosive" or "gender identity" or "sexual maturation" or "sexual relationship" or hyperkinetic or "sibling rivalry" or "stereotyped movement" or manic or "olfactory reference" or gaming or erectile or orgasmic or arousal or penetration or "sexual desire" or "bodily distress" or stress) adj4 (disorder* or syndrome*)).ab,kf,ti. 221064

23 ((substance* or drug* or caffeine or cannabi* or phencyclidine or inhalant* or opioid* or sedative* or hypnotic* or anxiolytic* or stimulant* or tobacco or cocaine or "volatile solvent*" or harmful or alcohol) adj3 (abuse* or use* or misuse* or addiction*)).ab,kf,ti. 431645

24 15 or 16 or 17 or 18 or 19 or 20 or 21 or 22 or 23 3148512

25 5 and 14 and 24 9087

26 limit 25 to (yr="2013 -Current" and (english or french)) 7085
