# Supplementary material for: The role of sociodemographic factors on the acceptability of digital mental health care: A scoping review protocol
Source: PLoS One. 2024 Apr 26;19(4):e0301886. doi: 10.1371/journal.pone.0301886 (PMC11051634; doi:10.1371/journal.pone.0301886)
Supplement: Attachment — Submitted filename: Letter_Response to Reviewers_V2_Feb29th_2024.docx [file pone.0301886.s004.docx]

We thank the reviewers for their constructive reviews and are encouraged to receive feedback that our revisions have “greatly improved the protocol”. We greatly appreciate the opportunity to submit a further revision of our paper for your continued consideration. Below, we respond to each reviewer’s comments and provide an overview of the corresponding tracked changes we made in the paper.

**Reviewer 1 Comment:**

1. I thank the authors for their responses to our comments and revision of the paper. I believe the authors’ efforts to address our comments greatly improve the protocol, and I am happy with the changes made.

**Our response:** We are happy to hear that the changes we have made are satisfactory. Thank you for the thoughtful feedback to help improve the manuscript.

**Reviewer 2 Comments:**

1. I have one suggestion related to the concept of 'acceptability', which is well defined in the paper, but less so in the search strategy which was included as an appendix. The terms 'patient preference', 'consumer satisfaction' and 'patient satisfaction' have not been included, and it seems these very similar - yet admittedly different - concepts may also yield important results. I'm wondering if including these terms in the first lines of the search along with the acceptability terms (lines 1-4) would be useful. If in testing these terms, they are not of use, perhaps a short explanation could be added? I am not confident that other authors will have as well defined a concept of acceptability and will be using other definitions.

**Our response**: We appreciate this suggestion very much. This concern also emerged as we were in the process of developing the search strategies for the present review. Our decision to omit these terms is based on the following: 1) First, the conceptualization of acceptability in this protocol is informed by the Technology Acceptance Model (TAM) and its two core components, perceived ease of use and perceived utility. In contrast to general satisfaction and preference, the TAM suggests, through its two core components, specific criteria to consider as one evaluates a given innovation. Despite the possibility of variations in the conceptualization of acceptability across studies, the general criteria used to evaluate acceptability based on the TAM should generally remain the same; 2) Furthermore, this decision was made to limit the scope of this review. Indeed, as evidenced by our search strategy, we have tried to be as inclusive as possible for the population and context component of this review. However, to ensure the feasibility of this review, this decision also meant that the concept component of the review would need to be scoped down. Our current strategy retrieved 12,894 articles (with duplicates removed) that will need to be screened – demonstrating already a broad approach. All this being said, we can appreciate how these decisions may impact our results. Therefore, we have added in the “limitation” section of the manuscript that the omission of these terms represents a limitation of this review.
